# Carqueja (*Baccharis trimera*) Protects against Oxidative Stress and *β*-Amyloid-Induced Toxicity in *Caenorhabditis elegans*

**DOI:** 10.1155/2015/740162

**Published:** 2015-07-07

**Authors:** Franciny Aparecida Paiva, Larissa de Freitas Bonomo, Patrícia Ferreira Boasquivis, Igor Thadeu Borges Raposo de Paula, Joyce Ferreira da Costa Guerra, Wagney Mendes Leal, Marcelo Eustáquio Silva, Maria Lúcia Pedrosa, Riva de Paula Oliveira

**Affiliations:** ^1^Núcleo de Pesquisa em Ciências Biológicas, Universidade Federal de Ouro Preto, Ouro Preto, Brazil; ^2^Departamento de Biodiversidade, Evolução e Meio Ambiente, Universidade Federal de Ouro Preto, Ouro Preto, Brazil; ^3^Departamento de Alimentos, Universidade Federal de Ouro Preto, Ouro Preto, Brazil; ^4^Departamento de Ciências Biológicas, Universidade Federal de Ouro Preto, Ouro Preto, Brazil; ^5^Departamento de Biologia Celular e Genética, Universidade Federal do Rio Grande do Norte, Natal, Brazil

## Abstract

Carqueja (*Baccharis trimera*) is a native plant found throughout South America. Several studies have shown that Carqueja has antioxidant activity *in vitro*, as well as anti-inflammatory, antidiabetic, analgesic, antihepatotoxic, and antimutagenic properties. However, studies regarding its antioxidant potential *in vivo* are limited. In this study, we used *Caenorhabditis elegans* as a model to examine the antioxidant effects of a Carqueja hydroalcoholic extract (CHE) on stress resistance and lifespan and to investigate whether CHE has a protective effect in a *C. elegans* model for Alzheimer's disease. Here, we show for the first time, using *in vivo* assays, that CHE treatment improved oxidative stress resistance by increasing survival rate and by reducing ROS levels under oxidative stress conditions independently of the stress-related signaling pathways (p38, JNK, and ERK) and transcription factors (SKN-1/Nrf and DAF-16/Foxo) tested here. CHE treatment also increased the defenses against *β*-amyloid toxicity in *C. elegans*, in part by increasing proteasome activity and the expression of two heat shock protein genes. Our findings suggest a potential neuroprotective use for Carqueja, supporting the idea that dietary antioxidants are a promising approach to boost the defensive systems against stress and neurodegeneration.

## 1. Introduction

Carqueja (*Baccharis trimera*) is a native plant found throughout South America. The plant has been used in popular alternative medicine to treat gastrointestinal and hepatic diseases as well as inflammatory processes and diabetes [1].

Several studies have shown that Carqueja has antioxidant activity* in vitro* [2, 3], as well as anti-inflammatory [2, 4, 5], antidiabetic [6], analgesic [5], antihepatotoxic [7], and antimutagenic [8] properties. More recently, Pádua et al. [9] showed that Carqueja also improves the* in vivo* antioxidant defense system in a rat inflammatory model induced by acetaminophen. Carqueja antioxidant activity has been related to its several phenolic compounds, including the polyphenols quercetin and rutin as well as phenolic acids such as caffeoylquinic acids [2, 3, 9, 10].

Phenolic compounds are widely found in various plant foods such as açaí [11], kiwi fruit [12], green tea, cocoa, and red wine [13], and they have many physiological and pharmacological functions.* In vitro* and* in vivo* studies have shown that phenolic compounds have powerful effects on biological responses by scavenging reactive oxygen species (ROS) and by activating cellular signaling pathways [11, 14]. Previous studies have also shown the capacity of phenolic acids to prevent damage in neuronal cells and suggest that phenolic compounds are efficient as neuroprotective agents [15]. Additionally, caffeoylquinic acids have been shown to protect against *β*-amyloid toxicity in MC65 and SHSY5Y neuroblastoma cell lines [16] and to have a neuroprotective effect in the mouse brain. These acids have also been shown to improve spatial learning and memory in senescence accelerated mice (SAMP8) [17].

Altogether, these findings have prompted health care professionals to recommend the ingestion of foods rich in naturally occurring antioxidants as a way to prevent chronic diseases, such as atherosclerosis, cancers, diabetes, and neurodegenerative disorders, and to promote health and well-being in general [18].

Even though a number of investigations have shown the antioxidant activity of Carqueja, very few of them explored its antioxidant potentials* in vivo.*

In this study, we used* Caenorhabditis elegans* as a model to examine the antioxidant effects of a Carqueja hydroalcoholic extract (CHE) on stress resistance, survival rate, and lifespan and to investigate whether CHE has a protective effect in a* C. elegans* model for Alzheimer's disease. Our study is the first to show that not only does Carqueja have antioxidant proprieties* in vivo*, but it also has protective effects against *β*-amyloid-induced paralysis in* C. elegans*. Our results also suggest that Carqueja's antioxidant proprieties are not mediated by the action of stress-related signaling pathways but are rather the result of a direct effect of CHE. Finally, CHE's potential neuroprotective action against *β*-amyloid toxicity could be, in part, due to increased proteasome activity and heat shock protein gene expression.

## 2. Materials and Methods

### 2.1. Chemicals and Reagents

Methanol (MeOH), DPPH (2,2-diphenyl-1-picryl-hydrazyl), Trolox (6-hydroxy-2,5,7,8-tetramethylchroman-2-carboxylic acid), Folin-Ciocalteu (FC) reagent, gallic acid,* tert*-butyl hydrogen peroxide (t-BOOH), and kanamycin were purchased from Sigma-Aldrich (St. Louis, MO, USA). 2′,7′-Dichlorodihydro-fluorescein diacetate (H_2_DCF-DA) and Nile red were purchased from Invitrogen (Eugene, Oregon, USA). Hydrogen peroxide (H_2_O_2_) was purchased from VETEC (Duque de Caxias, Rio de Janeiro, Brazil). Streptomycin was purchased from Serva Eletrophoresis.

### 2.2. Plant Material

The aerial parts of* Baccharis trimera* were collected from Mariana, Minas Gerais, Brazil. The collected plants were authenticated and a voucher specimen (OUPR 24050) was deposited at the Herbarium José Badini (UFOP). After identification, the aerial parts of the plant were dried in a ventilated oven or incubator, pulverized, and stored in plastic bottles. The preparation of Carqueja hydroalcoholic extract (CHE) was conducted according to Pádua et al. [3], with some modifications. One hundred g of the dried plant powder was extracted with distilled water and 70% ethanol 1 : 1 for 24 hours (h). Solids were removed by vacuum filtration and the solvent was removed by a rotary evaporator.

### 2.3. Worm Strains: Maintenance and Treatment

Nematodes used were the wild-type strain N2, BA17,* fem-1(hc17)*; EU1,* skn-1(zu67)IV/nT1*; CF1038,* daf-16(mu86)*; AU3,* nsy-1(ag3)*; KU4,* sek-1(km4)*; VC8,* jnk-1(gk7)*; AM1,* osr-1(rm1)*; KU25,* pmk-1(km25)*; MH37,* mpk-1(ku1)/unc-32(e189)*; MT2605,* unc-43(n498n1186)*; LD1171,* Is003 (gcs-1::GFP)*; CL2166,* dvls19[pAF15(gst-4::GFP::NLS)]*; CF1553,* muIs84 [pAD76(sod-3::GFP)]*; CL2070,* dvIs70[pCL25(hsp-16.2p::GFP)*+*pRF14(rol-6)]*; SJ4005,* zcls4[hsp-4::GFP]*; CL4176,* smg-1(cc546)*;* dvls27[pAF29(myo-3/Aβ*_*1–42*_ */letUTR)]*+*pRF4[rol-6(su10069)]* and TJ356,* zIs356[pGP30(DAF-16::GFP)+pRF4(rol-6)]*. All nematodes were cultivated on nematode growth medium (NGM) [19] at 20°C, except for the strain CL4176 which was maintained at 16°C. Synchronous L1 populations for all strains were obtained by hypochlorite treatment of gravid hermaphrodites except for* skn-1(zu67)* mutant and the transgenic lines expressing* gcs-1::GFP* (LD1171) and *β*-amyloid peptide 1–42 (CL4176) which were obtained by egg laying.

For all the experimental procedures, CHE was diluted in basal solution (0.1 M NaCl, 50 mM KPO_4_ buffer) to 0.5, 5, and 50 mg/mL and filter-sterilized. Basal solution (control) or CHE plus basal solution (treatment) was then mixed with an* E. coli* (OP50) pellet at OD600 of 1 and seeded onto NGM plates. In experiments conducted with dead bacteria, NGM plates seeded with* E. coli* OP50, with or without CHE, were treated with 10 mM kanamycin (KAN-treated).

RNA interference (RNAi) was carried out using the feeding method described previously, with empty pL4440 as the control [20]. Briefly, RNAi clones were grown with 12.5 *μ*g/mL tetracycline and 100 *μ*g/mL ampicillin. On the following day, cultures were diluted in LB supplemented with 60 *μ*g/mL ampicillin and grown to an OD600 of 1. This culture was used to seed plates containing ampicillin and 1 mM IPTG and left to dry for 1 day at room temperature. Synchronized L1 larvae were then placed at 20°C on* E. coli* HT115 that expressed* daf-16* and/or* skn-1* or control RNAi for 46 h, until they reached the L4 stage.* skn-1* RNAi efficiency was verified by the absence of F1 larvae. For* daf-16*, RNAi efficiency was confirmed by the suppression of GFP emission on* DAF-16::GFP* transgenic line (TJ356).

### 2.4. Polyphenol Dosage and DPPH Radical Scavenging Assay

Total polyphenol content of the CHE was determined by the Folin-Ciocalteu method as described by Georgé et al. [21]. In brief, 2.5 mL of Folin reagent was added to 500 *μ*L of sample or standard solution of gallic acid. After incubation at room temperature for 2 minutes (min), 2 mL of sodium carbonate 7.5% was added and incubated at 50°C for 15 min, after which the mixture was placed in an ice bath. Absorbance was determined at 760 nm. Total polyphenol content was expressed as gallic acid equivalents (GAE) per milliliter of extract. Two measurements were performed in triplicate and the mean values were calculated.

DPPH radical scavenging activity of CHE was determined as described by Brand-Williams et al. [22]. In short, 100 *μ*L of the CHE and standard Trolox antioxidant were added to 3.9 mL of 60 *μ*M DPPH dissolved in methanol 80%. The mixture was homogenized and incubated at room temperature in the dark for 30 min and the absorbance at 515 nm was determined. The percentage of inhibition was determined according to the formula = (1 − Abs Sample 515/Abs Control 515) × 100. The results were expressed as mean ± SEM (standard error of the mean) from 3 replicated measurements.

### 2.5. Bacterial Growth Assay

*E. coli* OP50 growth was evaluated over 5 h in the presence of CHE. All OD readings at 600 nm were normalized to the OD of the control group at time zero. Bacterial growth was measured in three individual experiments.

### 2.6. Body Length and Brood Size Assays

To measure body length, twenty L1 wild-type animals were treated or not with 50 mg/mL CHE until being 1-day-old adult and were then photographed using a Zeiss Axio Imager Z2 (NY, USA) microscope. Body length was determined by measuring along the animal axis using Axio Vision Rel. 4.8 software.

To assay brood size, ten L4 wild-type animals were individually transferred daily to new NGM plates containing or not 50 mg/mL CHE, until the completion of the egg laying period. The total progeny numbers for each plate were counted and divided by the number of animals. Three experiments were performed for both assays.

### 2.7. Pharyngeal Pumping Rate

Pharyngeal pumping rate was quantified using L4 wild-type animals treated or not with 50 mg/mL CHE since L1. For each condition, 10 animals were placed individually on agar pads with* E. coli* OP50 and allowed to acclimate for 5 min. Pharyngeal pumping was recorded for 2 min using an Olympus CX21 microscope (PA, USA) connected to a camera (Videolabs AmScope FMA050, MN, USA). The recording was digitized and slowed using Windows Movie Maker software (Microsoft Corp., 2002). The number of pumps was determined as an average of three independent counts for each animal over a time span of 20 seconds. A pump was defined as the movement of the pharyngeal grinder. This experiment was performed twice.

### 2.8. Lifespan and Oxidative Stress Assay

We used the strain* fem-1(hc17)* whose lifespan is similar to those of wild-type animals [23]. This strain is a temperature-sensitive mutant developing into semifertile adults at 20°C and sterile adults at 25°C which facilitates its growth and reproduction. The animals were synchronized at L1 stage and then maintained throughout their lifetime in plates containing or not 0.5, 5, and 50 mg/mL CHE. Approximately 90 animals were divided into 3 plates with 30 animals each. Nematodes were checked every day and scored as dead when they did not move even after repeated taps with a pick. Three experiments were performed.

Oxidative stress was induced by an acute, lethal concentration of* tert*-butyl hydrogen peroxide (t-BOOH) that is thermally more stable and less sensitive to contamination by metal than H_2_O_2_. About 50 worms treated or not with CHE on either regular or KAN-treated OP50 for 48 h were transferred to 7.5 mM t-BBOH while those treated or not with CHE on RNAi bacteria for 48 h were transferred to 12 mM t-BOOH. Viability was assessed after 6, 9, and 12 h. Three experiments were performed.

### 2.9. Measurement of Intracellular ROS in* C. elegans*

Intracellular ROS in* C. elegans* were measured using the fluorescent probe 2′,7′-dichlorodihydro-fluorescein diacetate (H_2_DCF-DA). Synchronized L1 wild-type animals were treated or not with 50 mg/mL CHE for 48 h and then transferred to 5 mM H_2_O_2_ or not for 1 h. Approximately 10 worms were collected into 1 mL of PBS with 1% Tween-20 (PBST), washed twice, and transferred into the wells of a 96-well microtiter plate containing 50 *μ*M H_2_DCF-DA. Samples were read every 30 min for 6 h in a multilabel microplate reader (Perkin Elmer VICTOR X3, MA, USA) at 37°C using excitation at 485 nm and emission at 535 nm. Three experiments were performed. Three measurements were performed in triplicate and the mean values were calculated.

### 2.10. Reporter Genes Analysis

The transgenic strains* gcs-1::GFP*,* gst-4::GFP*, and* sod-3::GFP* were treated or not with CHE for 48 h and then transferred or not to 7.5 mM t-BOOH for 1 h followed by a 1 h recovery period. The transgenic strains* hsp-16.2::GFP* and* hsp-4::GFP* were treated or not with 50 mg/mL CHE and then transferred or not to 35°C for 1 h followed by a 1 h and 30 min recovery period. Twenty worms from each group were photographed on a fluorescence microscope (Zeiss Axio Imager Z2, NY, USA) using a 10x ocular lens. GFP fluorescence signals were measured using NIH Image J software. Three experiments were performed.

### 2.11. Bioassays for *β*-Amyloid-Induced Paralysis

To determine if CHE suppresses or delays the onset of *β*-amyloid-induced progressive paralysis in CL4176 expressing muscle-specific A*β*1–42, freshly laid eggs were transferred to NGM containing 50 mg/mL CHE or control solution and incubated for 40 h at 16°C. To initiate amyloid-induced paralysis, the worms were up-shifted from 16°C to 25°C. Scoring was performed at 2 h intervals, typically after 24 h at 25°C. The worms were scored as “paralyzed” based either on the failure of the worms to move their body at the touch of a platinum loop or on the formation of a halo on the bacterial lawn, indicating a paralyzed condition. Each experiment was performed using at least 40 worms. These experiments were performed three times using living bacteria and two times using KAN-treated bacteria.

### 2.12. Proteasome Activity Quantification

*In vitro *26S proteasome activity assays were performed as described by Bonomo et al. [11]. Approximately 5,000 N2 wild-type animals were treated with control solution (S basal) or 50 mg/mL CHE for 48 h since L1. L4 worms were then harvested and sonicated. The lysates were centrifuged at 20,000 ×g for 20 min at 4°C. Protein extract was quantified using the QuantiPro BCA Assay Kit (Sigma Aldrich, St. Louis, MO, USA). To measure the chymotrypsin-like activity of the proteasome, the peptide succinyl-Leu-Leu-Val-Tyr-4-methyl-coumaryl-7-amide (SLLVY-MCA) (Sigma-Aldrich, St. Louis, MO, USA) was used in both presence and absence of 20 *μ*M MG-132, a proteasome inhibitor. Enzyme kinetics were monitored in a VICTOR X3 (Perkin Elmer, Massachusetts, USA) temperature-controlled microplate reader, every 15 min for 1 h at 37°C; the excitation and emission wavelengths were 380 and 460 nm, respectively. Relative proteasome activity was calculated as the difference between the total activity and the remaining activity in the presence of 20 *μ*M MG-132. The proteasome activity quantification was conducted three times.

### 2.13. Nile Red Staining

NGM plates were prepared containing 100 ng/mL Nile red and allowed to dry overnight at 37°C. Plates were seeded with* E. coli* OP50 mixed or not with 50 mg/mL CHE, and L1 worms were allowed to grow for 48 h. Twenty worms from each group were photographed on a fluorescence microscope (Zeiss Axio Imager Z2, NY, USA) using a 10x ocular lens. Fluorescence signals were measured using NIH Image J software. Three experiments were performed.

### 2.14. Statistical Analyses

Statistical analysis was performed using GraphPad Prism version 5.00 for Windows (CA, USA) and SAS JMP Statistical Discovery version 10.0 (NC, USA). The results were plotted as the mean ± SEM of three individual experiments. Data were subjected to the Kolmogorov-Smirnov test for normality. For data with a normal distribution, Student's *t*-test was used to compare pairs of groups, whereas a one-way ANOVA followed by Tukey's posttest was used to compare three or more groups. Nonparametric data were analyzed using the Mann-Whitney test when comparing two groups and the Kruskal-Wallis test followed by Dunn's posttest for comparing three or more groups. All survival curves were analyzed by the log-rank (Mantel-Cox) test. The statistical significance was determined as *P* < 0.05.

## 3. Results

### 3.1. The Carqueja Hydroalcoholic Extract (CHE) Tested Has* In Vitro* Antioxidant Capacity

To confirm that the CHE we produced has antioxidant properties, we quantified the total amount of polyphenols and measured the scavenging activity of the CHE through removal of the DPPH radical. The CHE total phenolic content was 0.085 ± 0.001 mg of gallic acid equivalent (GAE) per L of extract in hydroalcohol. We next determined the antioxidant capacity of 0.5, 5, and 50 mg/mL CHE by the DPPH radical scavenging activity method. We observed that CHE displays increasing* in vitro* antioxidant capacity in a dose-dependent manner (Table 1). At 50 mg/mL CHE, the highest concentration tested, the DPPH inhibition was 82.86%, which is equivalent to 700 mM Trolox, the reference standard, which shows 81.37% inhibition. We also evaluated the effect of bacteria metabolism on CHE's antioxidant capacity. The percentage of DPPH inhibition for 0.5 mg/mL CHE was reduced from 19.95% to 7.17% when the extract was mixed with* E. coli* OP50. Pretreating the bacteria with KAN before mixing with CHE restored the DPPH inhibition to 20.82% (Table 1). The percentage of DPPH inhibition for 5 and 50 mg/mL CH was not significantly altered when the extracts were mixed with bacteria (Table 1). These results indicate that bacteria metabolism interferes with the CHE antioxidant capacity at lower but not at higher concentrations of CHE.

### 3.2. CHE Increases Oxidative Stress Resistance in* C. elegans*

**Figure 1:**
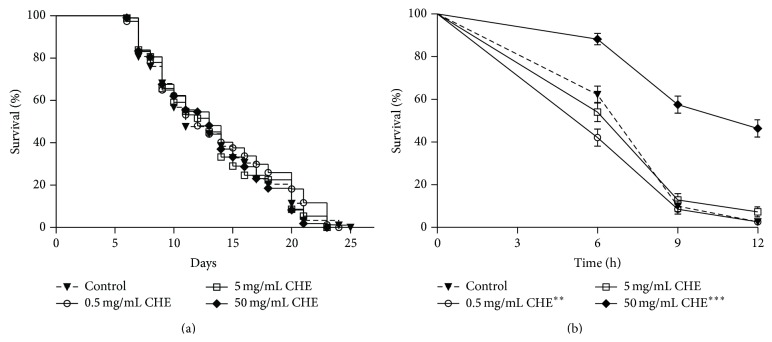
Effect of Carqueja hydroalcoholic extract (CHE) on* C. elegans* survival under standard laboratory and stress conditions. (a) Lifespan.* fem-1(hc17)* mutants animals were treated or not with three different CHE concentrations (0.5, 5, and 50 mg/mL) at 25°C beginning at L1. Nematodes were checked daily until all nematodes had died. Log-rank (Mantel-Cox) analysis showed no significant difference between the curves. (b) Stress resistance. Wild-type animals were treated or not with three different CHE concentrations (0.5, 5, and 50 mg/mL) from L1 to L4 and then submitted to 7.5 mM t-BOOH in M9. Survival was measured at 6, 9, and 12 h. The survival curves show that only 50 mg/mL CHE treatment increased* C. elegans* oxidative stress resistance, while 0.05 mg/mL CHE decreased oxidative stress resistance. ^***^ *P* < 0.001 and ^**^ *P* = 0.009 by the log-rank (Mantel-Cox) test.

We first tested whether CHE would affect the lifespan of* C. elegans* under standard laboratory conditions. Lifespan assays were performed in animals treated with three different CHE concentrations (0.5, 5, and 50 mg/mL) at 25°C. The mean lifespan of all treated animals did not differ significantly from controls lifespan (*P* = 0.42 for 0.5 mg/mL, 0.82 for 5 mg/mL, and 0.69 for 50 mg/mL) (Table 2). These findings indicate that, under standard conditions, CHE treatments at these concentrations did not alter* C. elegans* lifespan (Figure 1(a)).

It has been shown [2, 3, 9] that CHE has antioxidant activities* in vitro*; however, no* in vivo* oxidative models have been used to investigate these properties in animals under stress conditions. To explore whether CHE antioxidant properties have a protective effect* in vivo*, oxidative stress assays were performed in animals treated with three different concentrations of CHE (0.5, 5, and 50 mg/mL) for 48 h. We observed that animals treated with 50 mg/mL CHE showed increased oxidative stress resistance, when compared to controls (*P* < 0.0001). Surprisingly, animals treated with 0.5 mg/mL CHE showed a decreased oxidative stress resistance (*P* = 0.009), whereas in those treated with 5 mg/mL CHE there was no change in oxidative stress resistance (Figure 1(b), Table 3).

### 3.3. Increased Oxidative Stress Resistance Induced by CHE Remains in* C. elegans* Fed with Dead Bacteria

**Figure 2:**
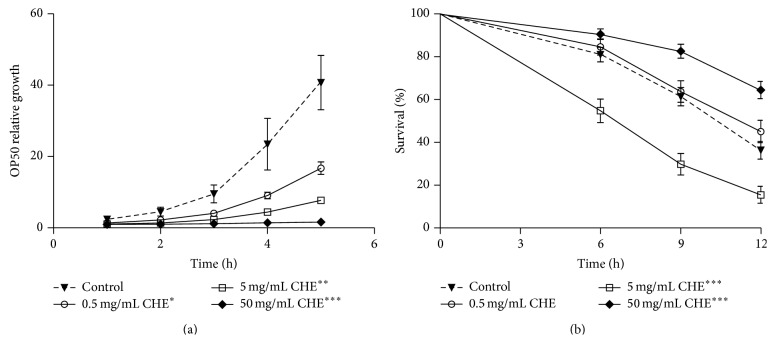
Effect of Carqueja hydroalcoholic extract (CHE) on* E. coli* growth and oxidative stress resistance of* C. elegans* fed with kanamycin-treated* E. coli*. (a)* E. coli* growth.* E. coli* OP50 growth was evaluated over 5 h in the presence of three different CHE concentrations (0.5, 5, and 50 mg/mL). The OD of the control group at time zero was used to normalize all other OD readings. ^*^Treatment of 0.5 mg/mL CHE decreased bacteria growth after 200 min. ^**^Treatment of 5 mg/mL CHE decreased bacteria growth after 120 min. ^***^Treatment of 50 mg/mL CHE decreased bacteria growth after 80 min. *P* < 0.05, determined by a two-tailed Student's *t*-test. (b) Stress resistance assay on bacteria killed with KAN. Wild-type animals were treated or not with three different CHE concentrations (0.5, 5, and 50 mg/mL) mixed with either* E. coli* OP50 or* E. coli* OP50 treated with 10 mM KAN from L1 to L4 and then submitted to 7.5 mM t-BOOH in M9. The survival was measured at 6, 9, and 12 h. The survival curves show that 50 mg/mL CHE treatment increased* C. elegans* oxidative stress resistance independent of its antibacterial effect. ^***^ *P* < 0.001 related to the respective controls by the log-rank (Mantel-Cox) test.

It is known that* E. coli* has a pathogenic effect on* C. elegans,* which affects its lifespan and stress resistance [24]. Therefore, we investigated whether the CHE protective effect we observed (Figure 1(b)) could result from inhibition of bacterial growth. When we assayed* E. coli* OP50 growth over 5 h in the presence of CHE, we observed an antimicrobial effect. This effect was observed after 80 min of incubation with 50 mg/mL CHE and after 120 and 200 min with 5 and 0.5 mg/mL CHE, respectively (Figure 2(a)).

However, in* C. elegans* fed with KAN-treated* E. coli,* treatment with 50 mg/mL CHE still increased the oxidative stress resistance (*P* < 0.0001), indicating that the CHE bacteriostatic propriety is not the only factor affecting oxidative stress resistance in these animals (Figure 2(b), Table 3). The treatment with 0.5 mg/mL CHE did not affect the oxidative stress resistance (*P* = 0.251) while with 5 mg/mL significantly decreased stress resistance (*P* < 0.0001). This result prompted us to perform the following assays using 50 mg/mL of CHE.

### 3.4. CHE Treatment Does Not Redirect Resources for Growth and Reproduction or Alter Feeding Behavior

**Figure 3:**
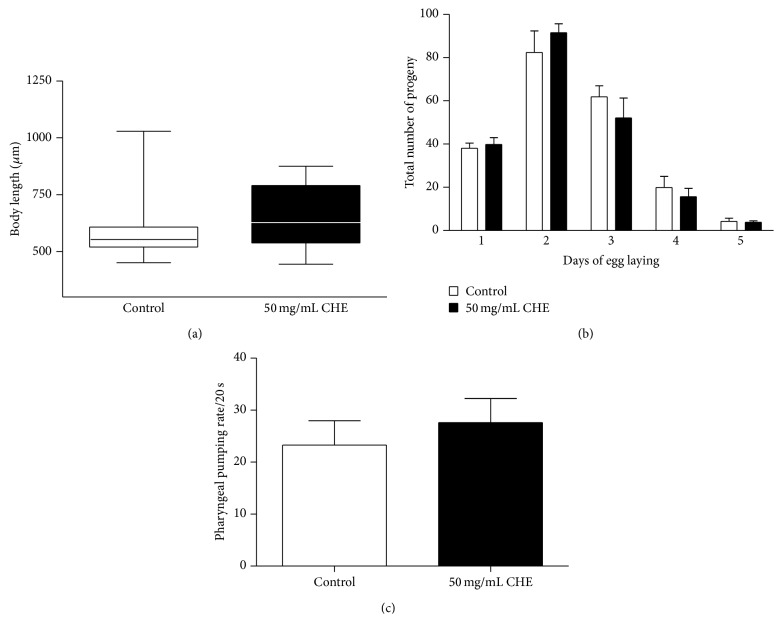
Effect of Carqueja hydroalcoholic extract (CHE) on wild-type* C. elegans* growth, reproduction and pharyngeal pumping. (a) Body length. L1 animals were treated or not with 50 mg/mL CHE for 48 h. Images were captured of L4 animals, and body length was measured along the animal axis using Axio Vision Rel. 4.8 software. There was no significant difference between groups, as determined by two-tailed Student's *t*-test. (b) Brood size. Progeny profiles were measured in animals treated or not with 50 mg/mL CHE. Animals were transferred individually to NGM plates and moved daily until the end of the reproductive period. The results were plotted as the mean ± SEM for each day. (c) Pharyngeal pumping. Pharyngeal pumping rate was quantified using L4 wild-type animals treated or not with 50 mg/mL CHE. There was no significant difference between groups, as determined by two-tailed Student's *t*-test.

It is known [25] that, in response to adverse environmental factors, energetic resources can be redirected to different developmental processes such as growth or reproduction. To verify whether CHE treatment would affect* C. elegans* growth and reproduction, we evaluated the body length and the progeny size of animals treated with 50 mg/mL CHE. The body length of treated animals was not significantly different from that of controls (*P* = 0.100) (Figure 3(a)). We did not observe any difference in the total progeny between controls and treated animals (*P* = 1.000) (data not shown). Moreover, 50 mg/mL CHE did not alter the egg-laying profile (Figure 3(b)). We also measured the pharyngeal pumping rate in L4 animals in order to verify whether CHE alters feeding behavior. The pumping frequency of 50 mg/mL CHE-treated animals was not significantly different compared to untreated animals (Figure 3(c)).

Taken together, these results suggest that CHE does not interfere with growth, reproduction, and food intake in* C. elegans*.

### 3.5. CHE Increases Oxidative Stress Resistance* In Vivo* by Reducing ROS

**Figure 4:**
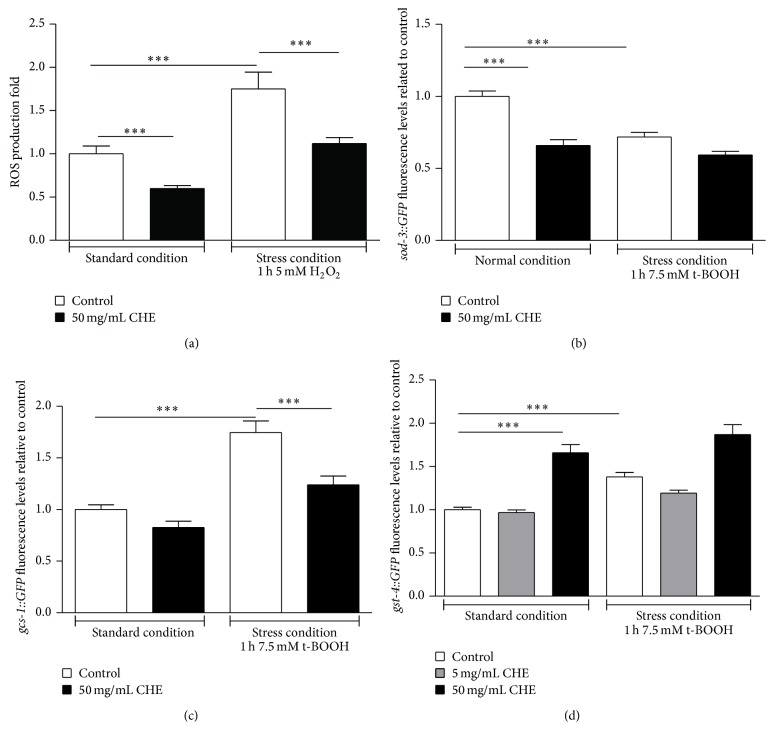
Effect of Carqueja hydroalcoholic extract (CHE) on redox status in wild-type* C. elegans.* (a) ROS production.* C. elegans* was treated or not with 50 mg/mL CHE for 48 h and then submitted to the presence or absence of 5 mM H_2_O_2_ for 1 h. CHE reduced ROS production under both standard and stress conditions. ^***^ *P* < 0.0001 by Kruskal-Wallis test followed by Dunn's posttest. Analysis of* sod-3::GFP* (b),* gcs-1::GFP* (c), and* gst-4::GFP* (d). Transgenic worms were treated or not with 50 mg/mL CHE for 48 h beginning at L1 and then with or without the oxidative stress condition. After a 1 h recovery period, photographs were taken on a fluorescence microscope and GFP fluorescence signals were measured using NIH Image J software. (b) Expression of* sod-3::GFP* is reduced in the 50 mg/mL CHE-treated group under standard conditions (c). CHE treatment reduced* gcs-1::GFP* expression under stress conditions. (d) Expression of* gst-4::GFP* increased in the 50 mg/mL CHE-treated group under standard conditions. ^***^ *P* < 0.0001 by Kruskal-Wallis test followed by Dunn's posttest.

Antioxidant phytochemicals can function either directly by scavenging ROS or indirectly through modulation of signaling pathways. To investigate whether the protective effect of 50 mg/mL CHE in treated* C. elegans* acts directly or indirectly, we measured ROS production and gene expression of* sod-3*,* gcs-1*, and* gst-4* antioxidant enzymes. CHE treatment reduced ROS production in animals under both standard and stress conditions (Figure 4(a)). CHE treatment reduced* sod-3::GFP* expression, if compared to untreated animals under standard conditions, but no significant differences were observed between treated and untreated groups under stress conditions (Figure 4(b)). Under stress conditions, CHE treatment reduced* gcs-1::GFP* expression, if compared to untreated animals, but no significant differences were observed between treated and untreated groups under standard conditions (Figure 4(c)). Expression of* gst-4::GFP* increased in the CHE-treated group, if compared to that observed in untreated animals under standard conditions. In animals under stress condition, no significant differences were observed between treated and untreated groups (Figure 4(d)). These results suggest that CHE treatment reduces ROS production and inhibits* sod-3::GFP* under standard conditions and* gcs-1::GFP* under stress conditions while it activates* gst-4::GFP* expression under standard conditions.

A number of genes and pathways have been identified in* C. elegans* to modulate lifespan and stress resistance. These highly conserved pathways include mitogen-activated protein kinase (MAPK) p38, JNK, and ERK MAPK pathways [26–29], which in turn may activate several transcription factors, including DAF-16 and SKN-1 [30–32]. To investigate whether these stress response signaling pathways would have a role in CHE-antioxidant activity, we performed the oxidative stress resistance assay in wild-type and mutant animals for DAF-16 and SKN-1 transcription factors and for the three MAPK pathways. For each stress-related signaling pathway, different mutants were tested (see Table 4 for details). All mutants treated with 50 mg/mL CHE showed increased oxidative stress resistance compared to their respective controls (Table 4). Since CHE has an antimicrobial effect on the bacteria, we repeated the stress assay for* nsy-1*,* jnk-1*, and* mpk-1* mutants that showed the smallest increase in median survival time. Still, even after being fed with CHE mixed with KAN-treated bacteria, these mutants exhibited increased oxidative stress resistance (Table 4). Therefore, the notion of possible antimicrobial effect induced oxidative stress resistance was rejected again. These results indicate that the oxidative stress resistance induced by CHE is neither mediated by these stress-related signaling pathways nor by the transcription factors tested here.

In order to rule out that a nonfunctional pathway could compensate by a functional one, we tested the double knockdown of SKN-1 and DAF-16 simultaneously. We observed that* skn-1(RNAi); daf-16(RNAi)* animals treated with 50 mg/mL CHE still showed increased oxidative stress resistance compared to their respective controls (Table 4).

### 3.6. CHE Treatment Delays Paralysis Induced by *β*-Amyloid (A*β*) Expression

The oxidative-stress state has an important role in the development of neurodegenerative diseases such as Alzheimer's (AD) and Parkinson's. Accumulation of ROS and deposition of toxic amyloid species have been proposed to exacerbate the symptoms observed in AD patients [33, 34].

In* C. elegans*, oxidative stress strongly correlates with A*β* toxicity, and it has been shown that a number of natural products that reduce ROS are neuroprotective [35, 36].

**Figure 5:**
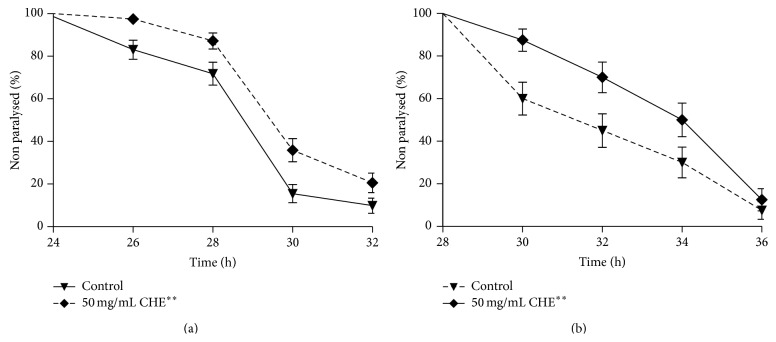
Effect of Carqueja hydroalcoholic extract (CHE) in transgenic* C. elegans* model for Alzheimer's disease. CL4176 transgenic strains treated or not with 50 mg/mL CHE were incubated for 40 h at 16°C. To initiate the *β*-amyloid-induced paralysis, the worms were up-shifted from 16°C to 25°C. The paralysis was verified each 2 h interval after 24 h at 25°C. The survival curves show that CHE alleviates *β*-amyloid-induced paralysis. (a) Paralysis profile of CL4176 transgenic animals fed with 50 mg/mL of CHE. ^**^ *P* = 0.0029 by the log-rank (Mantel-Cox) test. (b) Paralysis profile of CL4176 transgenic animals fed with 50 mg/mL of CHE mixed with KAN-treated bacteria. ^*^ *P* = 0.0353 by the log-rank (Mantel-Cox) test.

The observation that CHE has antioxidant properties* in vivo* led us to ask whether CHE treatment would have a protective effect against *β*-amyloid toxicity in a* C. elegans* model for the Alzheimer's disease. The expression of human A*β*1–42 in the muscle of transgenic CL4176 strain promotes a paralysis that can be monitored over time. We observed that the onset of paralysis was significantly delayed in 50 mg/mL CHE-treated animals (*P* = 0.0029). We observed that, after 24 h of induced A*β*1–42 expression, all CHE-treated animals were responsive to touch. After 32 h, this effect was still observed in 20% of the CHE-treated animals, compared to only 9% of the animals in the control group (Figure 5(a)). This effect was also independent of CHE antimicrobial effect. After 36 h, 50% of the CHE-treated animals were still responsive to touch, compared to 30% of the animals in the control group (*P* = 0.0353) (Figure 5(b)).

**Figure 6:**
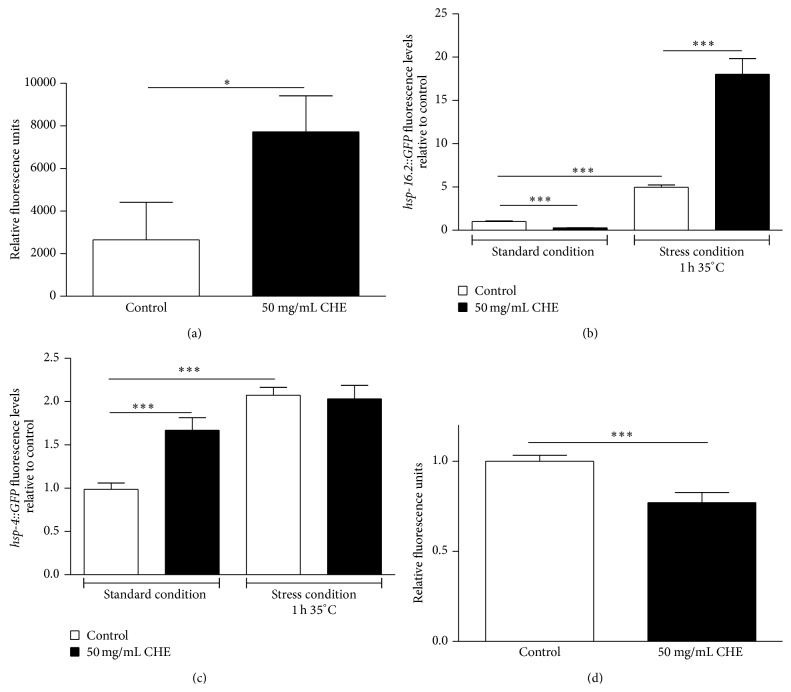
Effect of Carqueja hydroalcoholic extract (CHE) in protein homeostasis and Nile red staining. (a) Proteasome activity is increased by CHE treatment. Animals were treated or not with 50 mg/mL CHE from L1 to L4. Proteasome chymotrypsin-like activity was monitored by SLLVY-MCA digestion in L4 worm extracts containing equal amounts of total protein. ^***^ *P* < 0.05 by two-tailed Student's *t*-test. Transgenic worms* hsp-16-2::GFP* (b) and* hsp-16-2::GFP* (c) were treated or not with 50 mg/mL CHE for 48 h beginning at L1 and then transferred or not to 35°C for 1 h. After a 1 h and 30 min recovery period, photographs were taken on a fluorescence microscope and GFP fluorescence signals were measured using NIH Image J software. (b) CHE treatment increased* hsp-16-2::GFP* expression under stress conditions. (c) Expression of* hsp-4::GFP* increased in the 50 mg/mL CHE-treated group under standard conditions. ^***^ *P* < 0.0001 by Kruskal-Wallis test followed by Dunn's posttest. (d) Nile red accumulation reduction induced by CHE. Wild-type animals treated or not with 50 mg/mL CHE for 48 h beginning at L1 were maintained in plates containing Nile red. Subsequently, photographs were taken on a fluorescence microscope and GFP fluorescence signals were measured using NIH Image J software. CHE treatment reduced lipid fat deposit. ^***^ *P* < 0.0001 by Mann-Whitney test.

The disruption of protein homeostasis underlies the pathologies of most neurodegenerative disorders [37]. To evaluate whether CHE treatment alters protein homeostasis, we measured proteasome activity in animals treated with 50 mg/mL CHE. Proteasome chymotrypsin-like activity was monitored by SLLVY-MCA digestion in L4 worm extracts containing equal amounts of total protein. CHE increased proteasome degradation activity by 5-fold relative to the controls (*P* < 0.05) (Figure 6(a)).

Increasing the expression levels of heat shock proteins (HSPs) is known to enhance stress resistance and have a neuroprotective effect in* C. elegans* [38, 39]. Here, we tested whether 50 mg/mL CHE treatment could modulate the gene expression of* hsp-16.2* and* hsp-4* heat shock proteins. Under standard conditions, CHE treatment reduced* hsp-16.2::GFP* expression if compared to that observed in untreated animals. Animals under stress conditions showed increased* hsp-16.2::GFP* expression in the treated versus the untreated group (Figure 6(b)). Expression of* hsp-4::GFP* increased in the CHE-treated group, if compared to that observed in untreated animals under standard conditions. In animals under stress condition, no significant differences were observed between treated and untreated groups (Figure 6(c)).

Interestingly, HSP-4 is an essential endoplasmic reticulum (ER) chaperone that senses nutritional status and stimulates the expression of inducible lipases [40]. This activation is correlated with reduced levels of lysosome-related organelles (LROs) [40]. LROs are the site of concentration of lipophilic Nile red dye as well as age-associated autofluorescence. Therefore, we investigated whether CHE treatment would also cause LRO Nile red reduction. We observed that animals treated with 50 mg/mL CHE showed lower levels of Nile red fluorescence when compared to untreated animals (Figure 6(d)).

These results suggest that CHE treatment increases the defenses against A*β* toxicity by increasing proteasome activity and chaperone expression. Moreover, the increased* hsp-4* expression induced by CHE is associated with reduced LRO Nile red fluorescence.

## 4. Discussion

In popular medicine, infusions of the aerial parts of Carqueja are commonly used to treat gastrointestinal, liver, and a variety of inflammatory diseases [41]. Carqueja has been widely studied for its anti-inflammatory, antidiabetic, analgesic, antihepatotoxic, and antimutagenic properties [2, 4–8]. Although it contains the polyphenols quercetin and rutin as well as phenolic acids such as caffeoylquinic acids, there are no descriptions of its use against neurodegenerative disorders. Here, we show, for the first time, using* in vivo* assays, that treatment with CHE not only improved oxidative stress resistance by increasing survival rate and reducing ROS levels under oxidative stress conditions, but also increased defense against A*β* toxicity in* C. elegans*.

Our results show that CHE has* in vitro* (Table 1) and* in vivo* (Figure 1(b), Table 3) antioxidant properties, which were assessed through DPPH scavenging analysis and stress resistance assays. DPPH scavenging activity with 0.5 mg/mL CHE was low (19.95%). With 5 and 50 mg/mL CHE, higher antioxidant activities were found (74.27% and 82.86%, resp.). We also observed that bacteria metabolism interferes with the CHE antioxidant capacity at 0.5 mg/mL but not at 5 and 50 mg/mL (Table 3). Only the 50 mg/mL CHE treatment improved the survival rate of animals under stress (Figure 1(b)). Grünz et al. [42] showed by chemical and fluorescence analyses that flavonoids, such as quercetin, are taken up by* C. elegans* and are metabolized through conjugation in a dose-dependent manner. These findings may explain the fact that animals treated with lower CHE doses (0.5 and 5 mg/mL) failed to exhibit increased stress resistance (Figure 1(b)).

It is well known that bacteria used as a food source for* C. elegans* can be pathogenic and therefore may affect the animal's lifespan and response to oxidative stress [43]. Indeed, it has been suggested that nematodes may experience health benefits if the food source is composed of bacteria in conjunction with antimicrobial substances [44]. In this study, we observed that the 50 mg/mL CHE treatment improved the survival rate of* C. elegans* under stress even when the bacterial source was previously treated with KAN, indicating that the observed higher survival rate was not fully caused by a bacteriostatic effect of CHE (Figure 2(b)). Antimicrobial effects were also observed when working with isolated polyphenols and açaí extract [11, 44] without compromising the beneficial properties of these compounds in* C. elegans*. These findings strongly support an antioxidant effect for CHE itself.

Studies done in several animal models have shown a strong correlation between oxidative stress resistance and increased lifespan, which can be explained by a reduction in oxidative damage to biomolecules [42, 45, 46]. Although CHE increased stress resistance, we did not observe increased lifespan (Figure 1(a)). This lack of association between stress resistance and lifespan has been reported by other studies showing that the antioxidant effect is not predictive of increased lifespan [42, 46]. Although CHE did not increase lifespan, the fact that CHE did not interfere with growth and reproduction indicates that CHE is not toxic to* C. elegans* (Figures 3(a) and 3(b)).

The fact that ROS production was lower in* C. elegans* treated with CHE under stress and standard conditions suggests that the radical scavenging properties of CHE's phenolic compounds directly neutralize ROS production (Figure 4(a)). Moreover, we observed that CHE treatment reduced the expression of* sod-3::GFP* under standard conditions (Figure 4(b)). The radical scavenging activity of CHE probably leads to an improvement of cellular oxidative burden resulting in a repression of SOD-3. Quercetin treatment of* C. elegans* resulted in similar decreased expression of* sod-3::GFP* [47]. Cells under stress conditions use glutathione (GSH), the major cellular redox agent, in order to counteract the increased ROS production [48, 49]. The fact that the expression of* gcs-1::GFP* (*γ*-glutamylcysteine synthetase), which is the enzyme that synthetizes GSH, was lower under stress conditions in CHE-treated animals indicates that CHE was able to avert the need to synthesize more GSH (Figure 4(c)).

Glutathione S-transferases (GSTs) are considered one of the major players in phase II detoxification of both endogenous products of oxidative stress and electrophilic xenobiotics [50]. GST binds with GSH and catalyzes GSH conjugation with target substrates in order to facilitate their excretion from the cell. Several authors have reported that* gst-4* expression is induced by oxidative stressors and phytochemicals [51, 52]. We observed that 50 mg/mL CHE, but not 5 mg/mL CHE, significantly increased the expression of* gst-4::GFP* under standard conditions (Figure 4(d)). These results suggest that CHE may be xenobiotic and therefore it may function as a mild stressor.

Taking together our data on DPPH inhibition analysis, oxidative stress assays, and* gst-4::GFP* expression, we hypothesize that CHE's antioxidant capacity is counteracted by its xenobiotic properties and that bacteria metabolism plays a role in it. First, the percentage of DPPH inhibition for 0.5 mg/mL CHE was reduced from 19.95% to 7.17% when the extract was mixed with* E. coli* OP50. Pretreating the bacteria with KAN restored the CHE DPPH inhibition to 20.82% (Table 1). This reduction could explain why stress resistance of 0.5 mg/mL CHE-treated animals compared to control group is decreased (Figure 1(b) and Table 3) but it is not altered when CHE is mixed with KAN-treated bacteria (Figure 2(b) and Table 3). The percentage of DPPH inhibition for 5 and 50 mg/mL of CHE was not altered (74.27% and 82.86%, resp.) when the extracts were mixed neither with living bacteria (71.38% and 85.35%, resp.) nor with KAN-treated bacteria (73.43% and 87.57%, resp.). Since the quantity of bacteria added to the extract was always the same (OD600 = 1), these results indicate that bacteria metabolism interferes with the CHE antioxidant capacity at lower but not at higher concentrations. Based on* gst-4::GFP* expression, our results also indicate that 50 mg/mL CHE may be xenobiotic but 5 mg/mL is not (Figure 4(d)). Moreover, the survival rate under stress conditions of animals fed with 5 mg/mL CHE was not significantly different from control group (Figure 1(b), Table 3) but it was actually significantly lower than that of controls when the animals received 5 mg/mL CHE mixed with KAN-treated bacteria (Figure 2(b), Table 3). These results suggest that the bacteria metabolism could be reducing any CHE's xenobiotic capacity at 5 mg/mL without altering its antioxidant content. Finally, it is possible that the antioxidant effect of 50 mg/mL CHE overcomes any potential xenobiotic effect that might be observed with lower doses of CHE (5 mg/mL), but not with the lowest dose tested (0.5 mg/mL). If this is true, one would expect that, at some dose between 5 mg/mL and 50 mg/mL, CHE proprieties would shift from xenobiotic to antioxidant. Further studies should shed light on this interesting hypothesis.

It is known that dietary antioxidants may work directly through ROS scavenging, or indirectly through activation of stress-related signaling pathways [15, 53]. Mitogen-activated protein kinase (MAPK) signal transduction pathways are conserved throughout eukaryotic animals [26–28]. These pathways transduce signals in response to a variety of extracellular stimuli, such as oxidative and thermal stress and exposure to heavy metals and drugs [52, 54, 55]. Here we individually tested at least one mutant for each of the three subgroups of MAPKs that have been identified, namely, extracellular signal-regulated kinase (ERK), c-Jun N-terminal kinase (JNK), and p38 kinases [26, 29] (see Table 4 for more details). We also tested individual mutants for two transcription factors (SKN-1 and DAF-16) and the double knockdown* skn-1(RNAi); daf-16(RNAi)* that are MAPKs pathways' targets (Table 2). SKN-1/Nrf is activated by the p38 and ERK MAPK pathways in response to oxidative stress or xenobiotics [31, 32]. DAF-16/FOXO has also been reported as a target of the JNK MAPK pathway [30]. Studies have shown the potential of antioxidants to activate either SKN-1 and DAF-16 in* C. elegans*, suggesting that these transcription factors mediate beneficial responses induced by antioxidants, such as increased oxidative stress resistance and lifespan [56, 57]. Considering that CHE-induced increased stress resistance was still observed in these mutants, our results suggest that none of these pathways is involved with this beneficial effect. Moreover, CHE-induced increased stress resistance was still observed in animals submitted to double knockdown of SKN-1 and DAF-16 simultaneously. This finding further supports a direct mode of action for CHE.

Alzheimer's disease (AD) is a neurodegenerative disorder characterized by formation of plaques composed primarily of insoluble A*β*. The toxic nature of A*β*1–42 makes it a marker of AD progression and a target for new therapeutic treatments. Epidemiological studies have reported that consumption of phenolic compounds may reduce the risk of developing AD [58]. Many compounds such as derivatives of quinic acid and polyphenols such as the flavonoid quercetin have been identified in Carqueja extracts of different polarities [10, 59]. Interestingly, various studies have shown that the protective effect of phenolic compounds, including quercetin and caffeoylquinic acids, against amyloid-induced neurotoxicity is due to their antioxidant properties and to reduced generation of toxic A*β* species [33, 60]. These studies agree with our findings in which CHE alleviates *β*-amyloid-induced paralysis in* C. elegans* (Figure 5). Therefore, this result might be explained by CHE antioxidant action.

Increased proteasome activity and chaperone activation are among the highly conserved responses employed by eukaryotic cells to maintain protein-folding homeostasis (proteostasis) [61, 62]. The failure of these mechanisms to adequately respond to proteotoxic imbalances results in the accumulation of misfolded proteins, which can lead to neurodegeneration diseases such as Alzheimer's. To investigate whether CHE could be involved with protein homeostasis, we evaluated proteasome activity and expression of the chaperone genes* hsp-16.2* and* hsp-4*. In* C. elegans*, HSP-4, an ER chaperone, plays a central role in response to stress associated with heat shock and protein misfolding [63]. HSP-16.2 has been shown to prevent unfolded proteins from aggregating and to interact with intracellular human A*β*1–42 peptide [64, 65]. We observed that 50 mg/mL CHE treatment increases proteasome degradation activity and* hsp-4::GFP* expression under standard conditions (Figures 6(a) and 6(c)). Moreover, we observed that CHE significantly increased the expression of* hsp-16.2::GFP* under heat stress conditions (Figure 6(b)). These results suggest that CHE alleviated *β*-amyloid-induced toxicity, in part, by increasing proteasome activity and expression of two heat shock protein genes.

HSP-4 also stimulates expression of fasting-induced lipases, which reduces LRO Nile red fluorescence upon fasting in* C. elegans* [40]. The observation that CHE treatment increased* hsp-4* expression prompted us to ask whether CHE treatment would reduce LRO Nile red accumulation. We observed that the level of LRO staining by Nile red was reduced when compared to untreated animals (Figure 6(d)). In* C. elegans*, LROs, like the lysosome, play a key role in trafficking of proteins and degradation products around the cell [66]. They are also the site of microscopic autofluorescence that accumulates as animals age [66, 67]. Therefore, a decrease in Nile red may serve as an indicator of increased stress resistance and lifespan, since the fluorescence is lower in long-lived mutants (e.g.,* daf-2*) compared to short-lived mutants (e.g.,* daf-16*) [68]. Our results are in agreement with this assumption, since CHE increased oxidative stress resistance (Figure 1(b)). Finally, our results suggest that the neuroprotective effect against A*β*1–42 induced by CHE is also a result of increased protein homeostasis.

## 5. Conclusions

Our data reveal that CHE improves the oxidative stress response of* C. elegans*, which is in line with previous* in vitro* findings [2, 3]. Our findings also suggest a potential neuroprotective use for Carqueja, supporting the idea that dietary antioxidants are a promising approach to boost defense systems against stress and neurodegeneration [55]. Characterization of the CHE tested here may provide additional information on its compounds and may help with a better understanding of how this powerful phytochemical works and how to use it to improve human health.

## Figures and Tables

**Table 1:** Carqueja hydroalcoholic extract (CHE) and Trolox antioxidant capacity *in vitro* by DPPH assay.

	% inhibition (mean ± SEM)		
	—	+ OP50	+ OP50 + KAN
CHE			
0.5 mg/mL	19.95 ± 0.25	7.17 ± 0.45	20.82 ± 0.05
5 mg/mL	74.27 ± 0.35	71.38 ± 0.29	73.43 ± 0.22
50 mg/mL	82.86 ± 0.25	85.35 ± 0.21	87.57 ± 0.71
Trolox^a^			
100 *μ*M	18.94 ± 0.46	n.d.	n.d.
300 *μ*M	38.05 ± 1.24	n.d.	n.d.
400 *μ*M	54.04 ± 0.46	n.d.	n.d.
700 *μ*M	81.37 ± 0.31	n.d.	n.d.

^a^Trolox, 6-hydroxy-2,5,7,8-tetramethylchroman-2-carboxylic acid.

^n.d.^Not determined.

**Table 2:** Effect of Carqueja hydroalcoholic extract (CHE) treatment on lifespan of *C. elegans fem-1 *strain at 25°C.

	Survival rate (days)			*P* versus control (log-rank)^b^	*n* ^a^
	Mean	Median	Maximal		
Control	13.09	11	18.1		88 (2)
0.5 mg/mL CHE	13.75	12	18.3	0.42	77 (2)
5 mg/mL CHE	13.08	13	19.5	0.82	93 (2)
50 mg/mL CHE	13.21	13	18.4	0.69	108 (2)

^a^Total number of animals analyzed. The number in parentheses indicates the number of independent trials.

^b^Comparisons were performed using the log-rank (Mantel-Cox) test.

**Table 3:** Effect of Carqueja hydroalcoholic extract (CHE) treatment on oxidative stress resistance of *C. elegans* wild-type fed or not with kanamycin-treated *E. coli* OP50.

	Mean survival rate (hours)	*P* versus control (log-rank)^b^	*n* ^a^
*E. coli* OP50			
Control	8.16		151 (3)
0.5 mg/mL CHE	7.52	0.0090	152 (3)
5 mg/mL CHE	7.98	0.9668	124 (3)
50 mg/mL CHE	10.37	<0.0001	153 (3)
*E. coli* OP50 + KAN			
Control	10.27		132 (3)
0.5 mg/mL CHE	10.45	0.251	91 (3)
5 mg/mL CHE	8.53	<0.0001	84 (3)
50 mg/mL CHE	11.19	<0.0001	138 (3)

^a^Total number of animals analyzed. The number in parentheses indicates the number of independent trials.

^b^Comparisons were performed using the log-rank (Mantel-Cox) test.

**Table 4:** Effect of 50 mg/mL Carqueja hydroalcoholic extract (CHE) treatment on oxidative stress resistance of *C. elegans* wild-type and mutants.

	Mean survival rate (hours)		*P* versus control (log-rank)^b^	*n* ^a^	
	Control	Treated		Control	Treated
*E. coli *OP50					
N2	9.6	10.8	<0.0001	669 (13)	645 (13)
*nsy-1(ag3) *	7.6	8.8	<0.0001	154 (3)	160 (3)
*pmk-1(ku25) *	7.3	8.7	<0.0001	152 (3)	154 (3)
*sek-1(km4) *	7.1	9.0	<0.0001	147 (3)	158 (3)
*skn-1(zu67) *	6.5	9.6	<0.0001	131 (3)	133 (3)
*daf-16(mu86) *	8.2	10.0	<0.0001	159 (3)	155 (3)
*jnk-1(gk7) *	9.0	9.9	<0.0001	153 (3)	152 (3)
*osr-1(rm-1) *	8.4	9.7	<0.0001	154 (3)	154 (3)
*unc-43(n498n1186) *	8.5	10.4	<0.0001	149 (3)	130 (3)
*mpk-1(ku1) *	7.5	9.3	<0.0001	158 (3)	161 (3)
N2 *control(RNAi) *	10.45	11.26	<0.0001	146 (3)	150 (3)
N2* skn-1(RNAi)*; *daf-16(RNAi) *	8.12	10.40	<0.0001	134 (3)	146 (3)
*E. coli *OP50 + KAN					
N2	10.32	11.15	<0.0001	306 (6)	283 (6)
*nsy-1(ag3) *	9.39	10.67	<0.0001	100 (2)	99 (2)
*jnk-1(gk7) *	9.89	10.51	0.0024	98 (2)	99 (2)
*mpk-1(ku1) *	8.64	9.83	0.0014	76 (2)	94 (2)

^a^Total number of hermaphrodites analyzed. The number in parentheses indicates the number of independent trials.

^b^Comparisons were performed using the log-rank (Mantel-Cox) test.
